# A Time–Frequency Domain Analysis Method for Variable Frequency Hopping Signal

**DOI:** 10.3390/s24196449

**Published:** 2024-10-05

**Authors:** Zhengzhi Zeng, Chunshan Jiang, Yuanming Zhou, Tianwei Zhou

**Affiliations:** 1 National Key Laboratory of Electromagnetic Space Security, Jiaxing 314000, China; 2 The 36th Research Institute of CETC, Jiaxing 314000, China

**Keywords:** radio monitoring, variable frequency hopping (VFH) signal, time–frequency domain

## Abstract

A variable frequency hopping (VFH) signal is a kind of frequency hopping (FH) signal that varies both in frequency and dwell time. However, in radio surveillance, the existing methods for unidentified signals using VFH cannot be effectively handled. In this paper, we proposed an improved joint analysis method based on time–frequency domain features, which adopts multi-level processing to solve the time–frequency domain feature analysis problem of the VFH signal. First, the received signal is pre-processed by Short-Time Fourier Transform (STFT) and binarization, and a highly discriminative time–frequency image is obtained; then, the fixed frequency signal is removed based on the feature of connected domains, and the conventional frequency hopping (CFH) signal is removed by density-based spatial clustering of applications with noise (DBSCAN); finally, the overlapping region is cropped by the joint energy peak time–domain continuity properties. After the above multi-level joint processing method, the problem of VFH signal processing is effectively solved. The simulation result shows that the Mean Square Error (MSE) between the output results and the time–frequency image of the original VFH signal tends to be close to 0 when the Signal-to-Noise ratio (SNR) is 5 dB.

## 1. Introduction

Most wireless communications in life use fixed frequency signals, which are sometimes subject to electromagnetic interference from other signal transmitting sources [1,2], resulting in degradation of communication quality or even interruption of communication, while the emergence of frequency hopping signals effectively solves the problem of communication susceptible to interference [3,4]. Frequency hopping communication extends the bandwidth by carrying information on different frequencies [5] for time-shared transmission, thus improving the anti-interference ability, which has been more widely and historically applied in the fields [6] of unmanned aerial vehicles, mobile communications and indoor wireless communications. To further improve the anti-interference ability, variable frequency hopping (VFH) communication [7,8] emerged, based on frequency hopping communication which increases the hopping time change rule, that is, the duration of each hop carrier frequency also changes randomly along with a kind of pseudo-random sequence [9,10], to achieve the effect that the carrier frequency and the hopping time both change and at the same time, this also increases the difficulty of radio monitoring [11,12]. In the future, as more civilian drones and other remote-controlled devices, modified radios and the like use the VFH communication system to take up spectrum resources, it will greatly increase the difficulty of monitoring and countering unidentified signals in key areas such as airports and government offices.

The research around frequency hopping signal analysis methods has been a hot topic in China and abroad; for example, ref. [13] uses a single channel receiver to achieve frequency hopping signal sorting by a signal arrival time sequence; ref. [14] uses an improved K-Harmonic Means (KHM) clustering algorithm to improve the efficiency of frequency hopping signal sorting; and [15] adopts a method based on a periodic parameter K-means clustering algorithm for multi-frequency hopping signals. However, all the above studies pertain to frequency hopping signals with no change in the hopping period, that is, conventional frequency hopping (CFH) signals, and for VFH signals, these algorithms are no longer applicable, and only some of the steps are of significance. At present, there is little literature on the analysis of VFH signals. Wang Miao et al. have carried out a series of studies [16,17] on the acquisition of VFH signals based on the method of blind source separation (BSS) [18,19], but it requires multiple sensors to receive and process the array signal. In light of the aforementioned issues, this paper is founded upon the time–frequency domain of the signal and engages with it at multiple levels. The methodology adopted does not require a priori information and it is relatively intuitive to obtain the important characteristics of the VFH signal in the time–frequency domain. This can assist in the identification of unidentified signals that may be occupying spectrum resources in radio monitoring.

## 2. Signal Model and System Description

Conventional frequency hopping signals control the carrier frequency by a pseudo-random sequence that jumps over time, thus allowing frequency bandwidth expansion. Its mathematical model [20] is

(1) $$s_{CFH}(t)=a(t)\sum_{k=1}^{L}\left[e^{\left(2\pi f_{k}t+\varphi_{k}\right)}g_{T}(t-kT)\right]$$

where a(t) is the complex envelope of the baseband signal; L is the number of hops; $f_{k}$ is the carrier frequency of the kth hop; $\varphi_{k}$ is the phase of the kth hop; T is the hopping period; and $g_{T}(t-kT)$ is the gate function.

**Figure 1:**
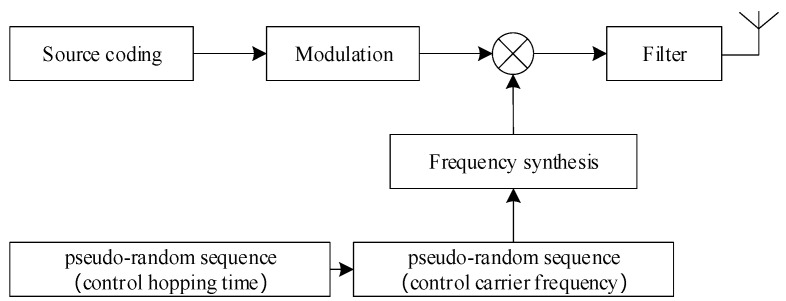
Basic block diagram of VFH signal generation.

The VFH signal adds a pseudo-random sequence to control the dwell time of each hop so that the carrier frequency changes as the hopping time changes. Its basic block diagram is shown in Figure 1.

Its mathematical model [16] is

(2) $$s_{VFH}(t)=a(t)\sum_{k=1}^{L}\left[e^{\left(2\pi f_{k}t+\varphi_{k}\right)}g_{T_{k}}(t-kT_{k})\right]$$

where $T_{k}$ is the frequency hopping period of the kth hop; the other parameters have the same meaning as in $s_{CFH}(t)$.

In this paper, we consider a mixed signal consisting mainly of a VFH signal, a CFH signal and a fixed frequency signal. Therefore, the model of the received signal is

(3) $$x(t)=s_{VFH}(t)+s_{CFH}(t)+s_{FF}(t)+w(t)$$

where $s_{FF}(t)=a(t)e^{\left(2\pi f_{c}t+\varphi (t)\right)}$ is a fixed frequency signal; w(t) is Gaussian white noise.

The algorithm for the analysis of the VFH signal is based on a time–frequency image. In this paper, Short-Time Fourier Transform (STFT) is used to perform the time–frequency transformation of x(t), which is defined [21] as follows:

(4) $$F_{STFT}(t,f)=\int x(\tau )h^{*}(\tau -t)e^{-j2\pi ft}d\tau$$

where h(t) is the window function and $h^{*}(t)$ is the conjugate function of the window function.

The time–frequency image is then binarized [22] to improve the contrast for the detection, identification and tracking of the time–frequency image features during the subsequent algorithmic analysis.

**Figure 2:**
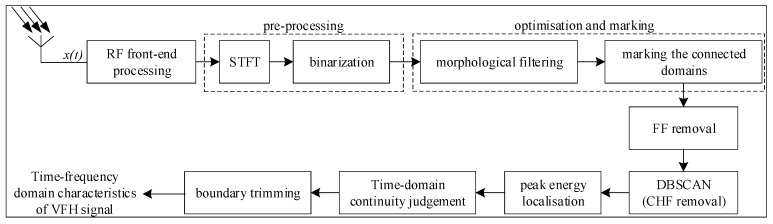
Block diagram of the VFH signal analysis system.

To summarise, consider the system description block diagram shown in Figure 2.

## 3. Design of Method for Analysing VFH Signal

### 3.1. Optimization Algorithm for Binarized Image Based on Morphological Filtering

When the STFT time–frequency image is binarized, a binarized image matrix I(m,n) with a scale of M×N is obtained, in which there may be some spurious points and there may also be holes or severe jaggies within the frequency-hopping region. The binarized image can be optimized by using morphological filtering; at the same time, because a fixed frequency signal has always been present, there is a clear time dimension continuity feature on the time–frequency domain, which can also be theoretically removed by this method.

Morphological filtering is based on set theory, which considers the image to be processed as set I, and constructs a set S to process set I. Among them, set S is called the structural element, which is an important factor in achieving the filtering. Morphological filtering has four basic operations [23]: dilation, erosion, open, and close, shown in Table 1.

Based on the above discussion, the specific procedure for optimising binarized images using morphological filtering is as follows:

Step 1. To eliminate spurious points, in the binarized image matrix I(m,n), for any 3 × 3 matrix, if all the elements of the surrounding eight neighbourhoods are 0 except the central element which is 1, then the central element is set to 0;

Step 2. To fill holes in the frequency hopping region and eliminate the jaggedness phenomenon, construct a rectangular structural element $S_{1}$ and perform the expansion operation on I(m,n) to obtain

(5) $$I_{1}(m,n)=I(m,n)⊕S_{1}=\{z:S_{1z}\cap I(m,n)\neq \emptyset \}$$

Step 3. To remove the fixed frequency signal, construct a linear structural element $S_{2}$ of length m. Perform the erosion operation on $I_{1}(m,n)$ to obtain a time–frequency image with only fixed frequency signal:

(6) $$I_{2}(m,n)=I_{1}(m,n)\Theta S_{2}=\{z:S_{2z}\subseteq I_{1}(m,n)\}$$

Step 4. Subtract $I_{2}(m,n)$ from the time–frequency image matrix $I_{1}(m,n)$ to obtain the time–frequency image matrix after removal of the fixed frequency signal:

(7) $$I_{3}(m,n)=I_{1}(m,n)-I_{2}(m,n)$$

After the above process, the binarized image is optimized and the fixed frequency signal is removed. However, sometimes the narrow edge of the fixed frequency signal remains in $I_{3}(m,n)$, which is caused by the erosion operation shrinking the edge of the fixed frequency signal in $I_{1}(m,n)$ and narrowing the width of the fixed frequency signal in $I_{2}(m,n)$, and at this time it is necessary to filter twice, or to perform the dilation operation on $I_{2}(m,n)$ and then perform the subtraction operation.

### 3.2. Fixed Frequency Signal Removal Algorithm Based on Length Difference of Connected Domains

A connected domain can be defined as a collection of pixels that are adjacent to one another and possess identical properties. Each pixel within a connected domain is adjacent to at least one other pixel in the region. The rules for determining adjacency are typically based on a four-concatenation (top, bottom, left, right) or eight-concatenation (top, bottom, left, right, top left, top right, bottom left, bottom right) approach. According to the judgement rules, the desired connectivity domains can be obtained and further processing of each connectivity domain can be achieved by marking.

Regarding the problem of incomplete removal of fixed frequency signal by morphological filtering, a method based on the length difference of connected domains is proposed. By marking the connected domains [24] on the time–frequency image and extracting the length parameter of each marked domain, it is obvious that the length of the fixed frequency signal is the longest, so the fixed frequency signal can be removed by setting the matrix of the marked region to 0.

**Figure 3:**
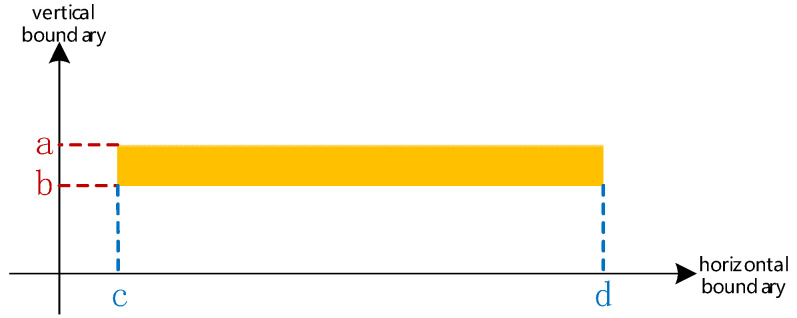
Four boundary values of the connected domain $Label_{FF}$.

The specific procedure is as follows:

Step 1. Label the connected domains in $I_{1}(m,n)$ to obtain the labelled set $Label_{i}(i=1,2,\ldots k)$, where k is the number of labelled connected domains;

Step 2. Extract length parameters from all the $Label_{i}$ to obtain the vector Label_length, and traverse to obtain the marker corresponding to the maximum value in Label_length, which is the marker $Label_{FF}$ of the fixed frequency signal;

Step 3. Extract the four boundary values a,b,c,d(a,b∈n;c,d∈m) from the connected domain $Label_{FF}$; See Figure 3.

Set the matrix of the region formed by the four boundary values to zero:

(8) $${I_{1}}^{\prime }(m,n)=I_{1}(c:d,a:b)=0$$

where ${I_{1}}^{\prime }(m,n)$ is the time–frequency image after removing the fixed frequency signal.

Compared to morphological filtering, this method does not require any secondary processing and can completely remove the fixed frequency signal; moreover, compared to the method of extracting the connected domain area parameter in the literature [25], if the bandwidth of the fixed frequency signal is very narrow and the bandwidth of the frequency hopping signal is large, there may be a situation where the area of a certain hop of the frequency hopping signal and the fixed frequency signal is equal. Therefore, the method in this paper is more accurate.

### 3.3. CFH Signal Removal Algorithm Based on DBSCAN Clustering

After removing the fixed frequency signal, only the CFH signal and the VFH signal remain, and to remove the CFH signal, the rule that its frequency hopping period is unchanged can be used. In the case of a few frequency hopping points, manual sorting can be used, but in practice, the amount of data collected is very large and machine sorting is needed; thus, the clustering algorithm can solve this problem.

One study [15,25] has used division-based clustering algorithms, including K-means and its improved algorithms, to classify frequency hopping points with similar characteristics into a cluster by specifying the number of nests and the clustering center, then iterating continuously. However, for the VFH signal, the frequency hopping period is not fixed and each frequency hopping point is considered a nest, which means that it cannot be considered as belonging to the same signal. Also, the more frequency hopping points there are, the more iterative clustering there will be, so it is no longer applicable.

DBSCAN clustering [26] is a density-based clustering algorithm which evaluates the similarity between data points by density, mainly based on the density-connected data points and a constant search for data points that satisfy the density condition, then clustering all the data points into different nests with the density condition. The period of each hop of the CFH signal is the same, and these points are connected based on the density, which satisfies the density condition and is clustered into one nest, while the period of almost every hop of the VFH signal is different, which does not satisfy the density condition and is clustered into another nest. The algorithm first needs to input a density condition: distance radius ε and the minimum number of points MinPts for the formation of a density circle; then, in the data set to be classified randomly in the selection of an unprocessed data point, if the data point as the center of the ε as the radius of the circle contains more than the number of points MinPts, then the point is the core object, and the core object and all its density accessible data points are classified as a nest. The process is repeated until all core objects have been traversed.

In light of the aforementioned discussion, the particular description of the CFH signal removal algorithm, based on DBSCAN clustering, is as follows:

Step 1. Use the length parameters of all connected domains in ${I_{1}}^{\prime }(m,n)$ as the input data set $\chi ={\left\{x_{i}\right\}}_{i=1}^{k-1}$ for DBSCAN clustering and set the clustering parameter (ε,MinPts);

Step 2. Calculate the density of each data point in χ:

(9) $$\rho_{i}=\left|\zeta_{\varepsilon }(x_{i})\right|$$

where $\zeta_{\varepsilon }(x_{i})=\left\{x_{j}\in \chi |d_{i,j}\leq \varepsilon \right\}$, and $d_{i,j}$ is the distance between $x_{i}$ and $x_{j}$;

Step 3. For any data point $x_{i}$ in $x_{i}$, if $\rho_{i}\geq MinPts$, it is considered to be a core point, and then the core point is extended: if $x_{j}\in \zeta_{\varepsilon }(x_{i})$, it is considered that $x_{j}$ is the density directly reachable from $x_{i}$, and in this way we can find any points that can be density connected from the core point;

Step 4. Core point confirmation and neighbourhood density linking are performed for the next data point in χ, and an iterative operation is performed until no core point is generated in χ, and all core points are classified as a nest $\Gamma_{1}$, while for the other points, they are classified as a nest $\Gamma_{2}$;

Step 5. $\Gamma_{1}$ corresponds to the length parameters of the connected domains of the CFH signal, and the matrices of the connected domains corresponding to each hop of the CFH signal are set to 0 using the operation of Equation (8) to obtain ${I_{1}}^{\prime \prime }(m,n)$.

After the above process, the CFH signal is removed. The VFH signal remains.

### 3.4. Overlapping Hopping Region Recovery Algorithm with Joint Energy Peak Time–Domain Continuity Properties

After DBSCAN clustering, sometimes a hop of the CFH signal will overlap with a hop of the VFH signal. These two hops are determined to be a connected domain when labelling is performed, so after removing most of the hops of the CFH signal, if the above overlap exists, it is also necessary to remove the CFH signal in the overlapping connected domain.

Due to the energy of the overlapping hopping region being significantly higher than other positions, the overlapping hopping region can be quickly located by the three-dimensional time–frequency image; secondly, because the VFH signal is continuous in the time dimension and the two hops before and after do not overlap, this property is used to crop the overlapping connected domain and remove the CFH signal. The specific description is as follows:

Step 1. Locate the overlapping hopping region by the energy peak of the three-dimensional time–frequency image, corresponding to the connected domain $Label_{overlap}$ in ${I_{1}}^{\prime \prime }(m,n)$;

Step 2. Assuming that the connected domain of the neighbouring hop overlapping with the time dimension of $Label_{overlap}$ is $Label_{neighb}$, extract its time dimension boundary value o(o∈m) on the side of $Label_{overlap}$, and then set the matrices belonging to others to o(o∈m) in the time domain of $Label_{overlap}$ to 0;

Step 3. Cut out the possible protruding parts of the vertical direction in the $Label_{overlap}$ and finally recover the VFH signal in the overlapping region.

### 3.5. Flowchart of the VFH Signal Analysis Method

**Figure 4:**
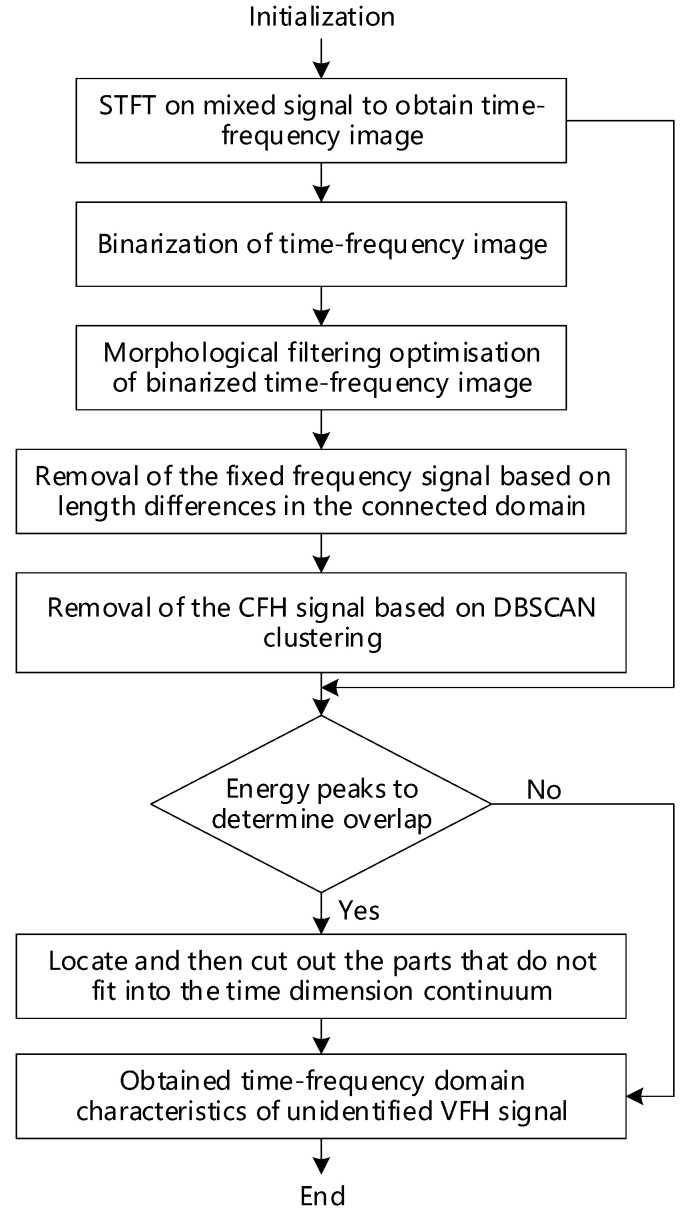
Flowchart of the VFH signal analysis method.

According to the previous theoretical analysis, the flowchart of the VFH signal analysis method in this paper is shown in Figure 4.

## 4. Simulation Results and Analysis

A mixed signal is constructed containing a VFH signal, a CFH signal, a fixed frequency signal and Gaussian white noise, with the relevant parameters shown in Table 2. Among them, the frequency and hopping period of the VHF signal are generated by constructing a random sequence; the frequency of the CHF signal is also generated by constructing a random sequence, but the hopping period is fixed at 50 μs for observation; other parameters can be set as desired.

**Figure 5:**
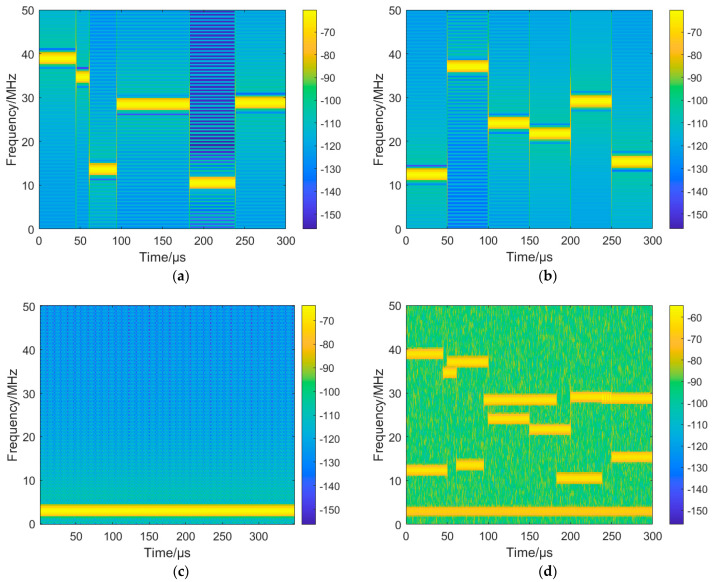
Time–frequency images of signal components and mixed signal. (**a**) VFH. (**b**) CFH. (**c**) Fixed frequency signal. (**d**) Mixed signal.

The time–frequency images of the signal components and the mixed signal are shown in Figure 5.

**Experiment 1.**  *Optimization on of Binarized Time–frequency Image Based on Morphological Filtering.*

**Figure 6:**
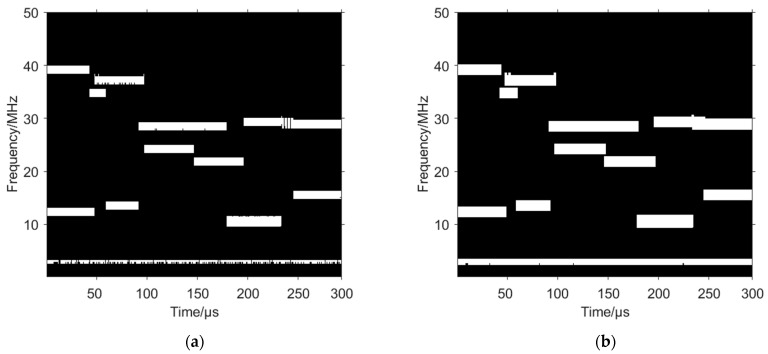
Optimization of binarized time–frequency image based on morphological filtering. (**a**) Binarized time–frequency image before optimization. (**b**) Optimized binarized time–frequency image.

The mixed signal is binarized to obtain the binarized time–frequency image shown in Figure 6a. Although there are no spurious points, there are more serious holes and jaggies, which will certainly affect the subsequent processing, so here a 5×4 rectangular structural element is constructed to perform the dilation operation on the binarized time–frequency image, which fills the holes and almost eliminates the jaggies, as shown in Figure 6b.

**Figure 7:**
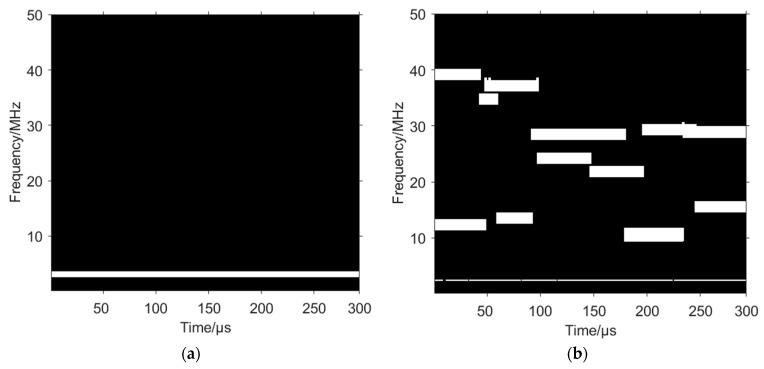
Morphological filtering to remove fixed frequency signal. (**a**) Fixed frequency signal extracted by erosion operation. (**b**) Time–frequency image obtained after subtraction.

The use of morphological filtering to remove the fixed frequency signal is then considered. Here, a linear structural element of length 600 is constructed and the erosion operation is applied to the binarized time–frequency image to extract the time–frequency image containing only the fixed frequency signal, as shown in Figure 7a, which is then subtracted from the original binarized time–frequency image to obtain Figure 7b. There is a narrow boundary due to the edge shrinkage of the fixed frequency signal caused by the erosion operation.

**Experiment 2.**  *Fixed Frequency Signal Removal Based on Length Difference of Connected Domains.*

**Figure 8:**
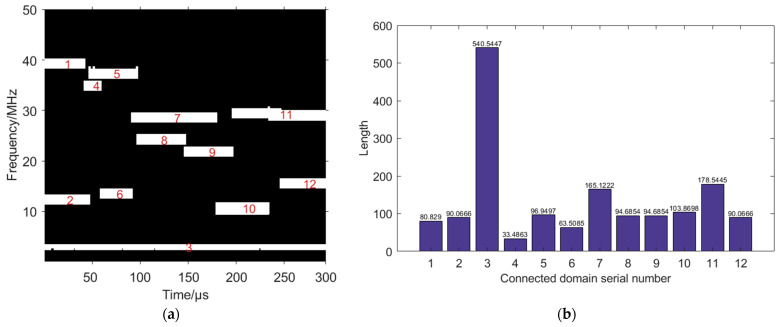
Connected domain labelling and length statistics. (**a**) Connected domain labelling. (**b**) Connected domain length statistics.

**Figure 9:**
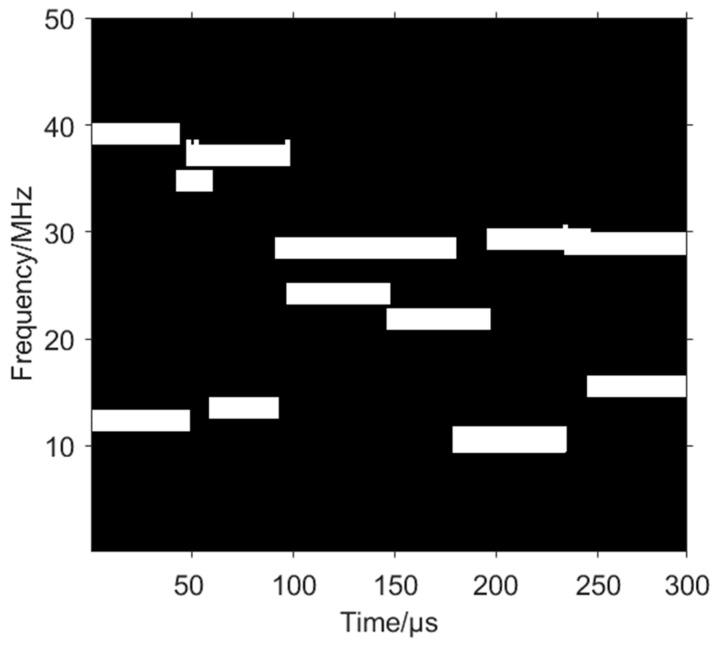
Fixed frequency signal removal based on length difference of connected domains.

The binarized time–frequency image is labelled with connected domains, as shown in Figure 8a, and then the length of each connected domain is counted, as shown in Figure 8b. The length of the third connected domain is greater than the lengths of the other connected domains, with a value of 540.5447, which corresponds to the fixed frequency signal, so the matrix corresponding to this connected domain is set to 0, thus removing the fixed frequency signal in its entirety, as shown in Figure 9.

**Experiment 3.**  *CFH Signal Removal Based on DBSCAN Clustering.*

**Figure 10:**
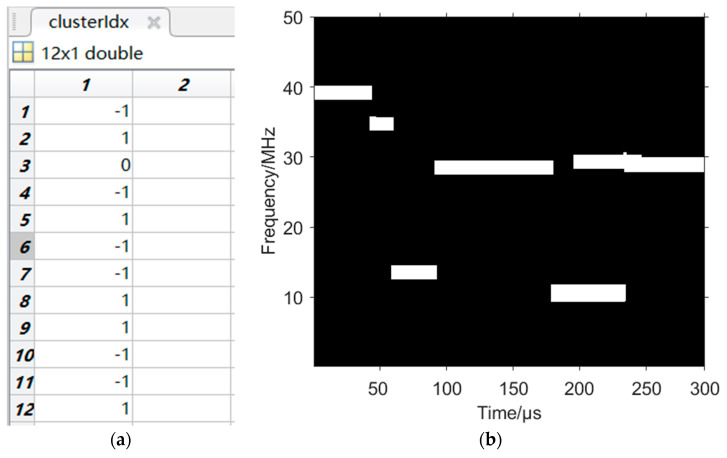
CFH signal removal based on DBSCAN clustering. (**a**) Clustering result parameter. (**b**) Time–frequency image with CFH signal removed.

The lengths of the connected domains in the time–frequency image after removing the fixed frequency signal are taken as the input data set, and then the density condition is discussed: because the frequency hopping period of each hop of the CFH signal is the same, there will not be a large error even after the above processing; therefore, the clustering parameter ε=5, MinPts=3 is set to obtain the result parameter as shown in Figure 10a, where {1,4,6,7,10,11}=−1 is a nest corresponding to the connected domains of the VFH signal; {2,5,8,9,12}=1 is a nest corresponding to the connected domains of the CFH signal. Then, the matrices corresponding to the connected domains of the CFH signal are set to 0 according to the method of Equation (8), and the time–frequency image is shown in Figure 10b.

At this point, most of the hops of the CFH signal have been removed and there is still one hop that overlaps with a hop in the VFH signal. If there is no such overlap, the VFH signal has been obtained.

**Experiment 4.**  *Overlapping Hopping Region Recovery for Joint Energy Peak Time–Domain Continuity Properties.*

**Figure 11:**
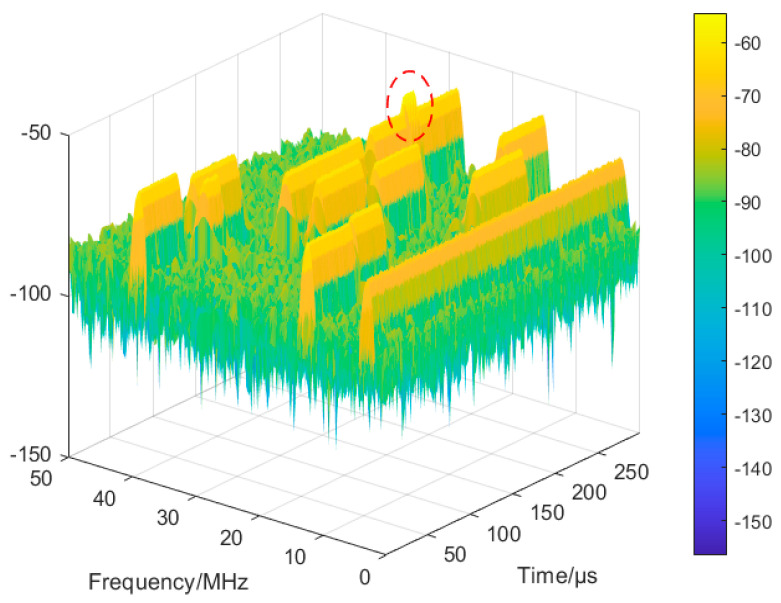
Energy peak in three-dimensional time–frequency image.

When the STFT is performed on the mixed signal, its three-dimensional time–frequency image is also observed and used as a reference after DBSCAN clustering. If there is no obvious energy peak, there is no hopping overlap, and the result after DBSCAN clustering sorting is the time–frequency image of the VFH signal; if there is an obvious energy peak, as shown in Figure 11, there is hopping overlap (corresponding to Figure 10b).

**Figure 12:**
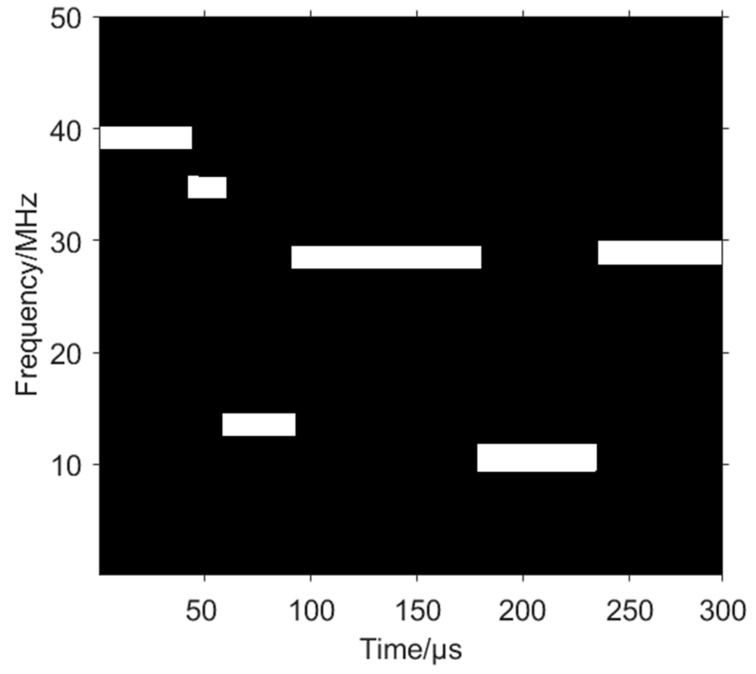
Sorted VFH signal.

Through the energy peak of the three-dimensional time–frequency image, to quickly locate the overlapping hopping region, use the time dimension continuity of each hop of the VFH signal, crop the part that conflicts with the neighbouring hop’s time dimension, crop the parts of the vertical direction as well to recover the hop of the VFH signal, and finally obtain the complete time–frequency image of the sorted VFH signal, as shown in Figure 12.

**Experiment 5.**  *Analysis of Algorithm Performance Under Different Signal-to-Noise Ratio (SNR) Conditions.*

**Figure 13:**
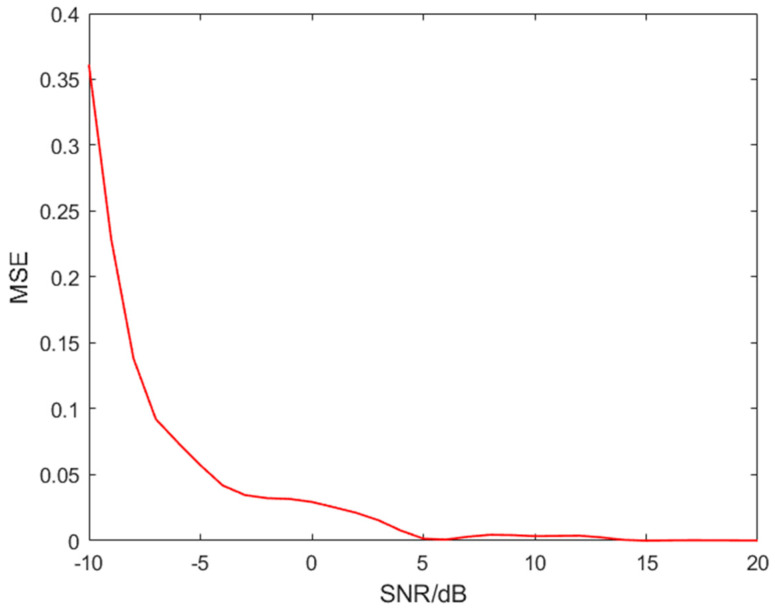
MSE under different SNR conditions.

To verify the performance of the algorithm, the result of the analysis of the mixed signal is compared to the original VFH signal, and the difference between the two time–frequency image results is analysed using the Mean Square Error (MSE) [27].

(10) $$MSE=\frac{1}{M\cdot N}\sum_{i=0}^{M-1}\sum_{j=0}^{N-1}{\left[I_{original}(i,j)-I_{output}(i,j)\right]}^{2}$$

where M is the number of matrix elements of the time–frequency image of the original VFH signal $I_{original}(i,j)$; N is the number of matrix elements of the mixed signal analysed result $I_{output}(i,j)$. With the SNR set to −10 dB~20 dB and the rest of the conditions unchanged, the Monte Carlo simulation is performed, and the results are shown in Figure 13.

It can be seen that when the SNR is 5dB, the MSE value is close to 0 and the time–frequency domain characteristics of the VFH signal can be obtained perfectly. The corresponding time–frequency images obtained from the actual analysis results are also the same.

## 5. Conclusions

The method proposed in this paper can be effective in radio monitoring to obtain important characteristics of the VFH signal that may occupy spectral resources. It is based on the STFT and binarization of the received mixed signal; the time–frequency image is optimized by morphological filtering; the fixed frequency signal is removed using the length difference in the connected domains; and then the CFH signal is removed using the DBSCAN clustering algorithm. In the case of hopping overlap, the top of the VFH signal in the overlapping hopping region is recovered by the signal energy peak and the continuous property of the VFH signal in the time dimension. The experimental results show that the above algorithmic process can effectively obtain the time–frequency domain characteristics of the VFH signal at an SNR of 5dB. There is little literature on the analysis of VFH signals, and the approach in this paper provides a new perspective on this analysis.

## Figures and Tables

**Table 1:** Four basic operations of morphological filtering.

Basic Operation	Definition	Function
Dilation	$I⊕S=\{z:S_{z}\cap I\neq \emptyset \}$	Boundary expansion; hole filling.
Erosion	$I\Theta S=\{z:S_{z}\subseteq I\}$	Boundary contraction; elimination of points smaller than structural element.
Open	I∘S=[IΘS]⊕S	Separate regions; eliminate points smaller than structural element.
Close	I•S=[I⊕S]ΘS	Connecting separate regions; filling in gaps.

where z is a point on set I.

**Table 2:** Simulation parameters.

Parameter	Description
Frequency set of VFH signal	[38.93, 34.65, 13.60, 28.45, 10.55, 28.80] MHz
Frequency hopping period of VFH signal	[44.87, 16.25, 33.32, 88.46, 55.50, 61.60] μs
Frequency set of CFH signal	[12.38, 37.08, 24.13, 21.73, 29.13, 15.38] MHz
Frequency hopping period of CFH signal	50 μs
Frequency of fixed frequency signal	3 MHz
Gaussian white noise	10 dB
Window function of STFT	128-point Hamming window
Sampling rate	100 MHz

## Data Availability

Due to the nature of this research, participants of this study did not agree for their data to be shared publicly, so supporting data is not available. However, some of the process elements of this study (non-critical data) are available from the corresponding author upon request.
